# Ability of MultiColor scanning laser ophthalmoscope to detect non-glaucomatous retinal nerve fiber layer defects in eyes with retinal diseases

**DOI:** 10.1186/s12886-018-0995-8

**Published:** 2018-12-17

**Authors:** Hiroto Terasaki, Shozo Sonoda, Naoko Kakiuchi, Hideki Shiihara, Takehiro Yamashita, Taiji Sakamoto

**Affiliations:** Department of Ophthalmology, Kagoshima University Graduate School of Medical and Dental Sciences, 8-35-1 Sakuragaoka, Kagoshima, 890-8520 Japan

**Keywords:** Non-glaucomatous NFLD, Scanning laser ophthalmoscope, Spectralis, MultiColor

## Abstract

**Purpose:**

To compare the ability of ocular fundus images obtained by Spectralis MultiColor scanning laser ophthalmoscope (MC-SLO) to that obtained by conventional color fundus images (CF) in detecting non-glaucomatous nerve fiber layer defects (NFLDs).

**Methods:**

A cross-sectional, retrospective study. Patients with retinal diseases who had ocular examination with both the MC-SLO and CF instruments at the Kagoshima University from December 2016 to February 2017 were studied. Eyes that had NFLDs with non-glaucomatous optic discs were analyzed. The visibility of the NFLDs was classified into three grades: grade 0, not visible; grade 1, barely visible; and grade 2, clearly visible. The NFLD grade for blue, green, and red scanning lights of the MC-SLO, merged images with three wavelengths and the color and red-free images were determined by two ophthalmologists. These scores were compared by Steel-Dwass tests.

**Results:**

Thirty-one eyes of 26 patients with a mean age of 63.1 ± 11.2 years were studied. There were 14 eyes with diabetic retinopathy, 11 eyes with age-related macular degeneration, 3 eyes with a branch retinal vein occlusion, and 3 eyes with an epiretinal membrane/macular hole. Both the intra-rater (0.631–0.790) and inter-rater (0.637–0.733) agreements were good. NFLDs were detected by the blue wavelength in all cases and by green wavelength and merged wavelengths in 90.3% of the images. The mean NFLD grade was 1.58 ± 0.49 for blue light images, 1.13 ± 0.54 for green light images, 0.07 ± 0.24 for red light images, and 1.16 ± 0.56 for merged images. The NFLD score for blue wavelength was significantly higher than that for green and red wavelength images (*P* < 0.05 and *P* < 0.01) but not significantly higher than that for the merged images. NFLDs were detected in 12 eyes (38.7%) in the color images and 16 eyes (51.6%) in the red-free images. The NFLD score for the CF and the red-free image was 0.41 ± 0.55 and 0.70 ± 0.67 which is significantly lower than that of blue MC-SLO images.

**Conclusion:**

The images obtained by MC-SLO are superior to that obtained by CF in detecting NFLDs in eyes with retinal diseases. We recommend MC-SLO imaging to screen for NFLDs in eyes with retinal diseases.

## Introduction

The Spectralis MultiColor scanning laser opthalmoscope (MC-SLO, Heidelberg Engineering, Heidelberg, Germany) is a newly developed instrument which uses three different wavelength laser scanning lights, viz., blue (488 nm), green (518 nm), and red (815 nm) [1]. The blue wavelength light does not penetrate deeper than the retinal nerve fiber layer and can be used to study the inner layers of the retina. The green wavelength scanning light penetrates into the retina and can be used to analyze the deeper layers of the retina, and the red wavelengths scanning light can reach the retinal pigment epithelium (RPE) and the superficial choroid and can be used to study the RPE and choroid. The merging of the images obtained by these three wavelengths should be able to detect findings from the different retinal layers much more clearly than conventional color fundus images. The resolution of each scan of this MC-SLO is 3.5 μm/pixel, thus it can record clearer and more detailed images than the conventional color fundus images. Several studies have shown the superiority of MC-SLO to other instruments that can obtain images of the different layers of the retina in normal and diseased eyes [1–3].

The retinal nerve fiber layer (RNFL) is the innermost layer of the retina and consists of the axons of the retinal ganglion cells (RGCs). The nuclei of the RGCs are located just distal to the RNFL [4]. It is well established that a thinning of the circumpapillary RNFL is a key feature of glaucomatous eyes [5], and nerve fiber layer defects (NFLDs) are representative findings in eyes with glaucoma. The NFLDs are seen ophthalmoscopically as dark-colored, slit-like areas running toward or touching the optic disc border, however they are difficult to see because the contrast between the NFLDs and the overall color of the fundus is not large. NFLDs can also be present in eyes with retinal diseases such as diabetic retinopathy, hypertensive retinopathy, and Behcet’s disease [6–9]. These changes are called “non-glaucomatous NFLDs” [7, 10], and special care is needed not to overlook these NFLDs because they are signs of retinal diseases which can be progressive [7]. In addition, the failure to detect these non-glaucomatous NFLDs in eyes with a normal appearing optic disc would tend to make clinicians to not perform additional examinations such as a perimetry and RNFL thickness analysis by optic coherence tomography (OCT). Because 50% of the RNFL can be damaged before they can be detected by conventional ophthalmoscopy [11], the detection of non-glaucomatous NFLDs is very important for the patients.

The purpose of this study was to compare the ability of MC-SLO images to that of conventional fundus images in detecting non-glaucomatous NFLDs in eyes with retinal diseases. To accomplish this, we examined images of the fundus obtained by MC-SLO to those obtained by conventional fundus photography in eyes with retinal diseases.

## Methods

### Ethics statement

All of the procedures used in this study conformed to the tenets of the Declaration of Helsinki. The procedures used were approved by the Ethics Committee of Kagoshima University Hospital.

### Subjects

This was a retrospective, cross sectional study that was performed at the Kagoshima University Hospital, Kagoshima, Japan. We initially reviewed the medical records of 297 eyes of 176 patients who had color fundus images obtained by a swept source optical coherence tomographic instrument (DRI OCT Triton, Topcon, Japan) and MC-SLO images obtained by Spectralis MultiColor scanning laser ophthalmoscope. The images were recorded on the same day between December 2016 and February 2017. The eyes that had NFLDs that were detected by two experienced ophthalmologists (SS, NK) in at least one of the MC-SLO or color fundus images, i.e., blue, green, red, or merged, of the MC-SLO, or in the color or red-free images were studied. For this examination, we did not use the enlargement function of the instruments and evaluated the original screen of the viewer attached to the instruments. When the interpretation was split between raters, the images were excluded. In addition, to investigate whether non-glaucomatous NFLD were observed in normal eyes in the MC-SLO and CF images, 46 eyes of 23 healthy subjects who did not have any ocular abnormalities in the ophthalmologic examinations were evaluated. The NFLDs appeared as dark slits or bands or diffuse darkness of the retina that passed toward or touched the optic disc for not more than 60° of the circumference [10]. Eyes with an IOP > 21 mmHg and eyes with optic discs that had glaucomatous changes such as generalized or focal enlargement of the cup, superficial splinter hemorrhage, development of vessel overpass were excluded. To include our subjects appropriately, the evaluations of the optic disc was done by two experienced ophthalmologists. Eyes with poor quality in MC-SLO images or color fundus images were also excluded.

### Comparison of NFLD scores

The visibility of NFLDs was divided into three grades: grade 0, not visible; grade 1, barely visible; and grade 2, clearly visible and defined as the “NFLD scores”. The NFLDs in the blue, green, red, and merged MC-SLO images were graded and also in the color and red-free images by two graders (HT, HS). In our preliminary experiments, the NFLDs tended to be more detectable in the MC-SLO images than in the CF images. Thus, the order of examination for the NFLDs was always begun with the color fundus images followed by the red-free images and then the MC-SLO images.

The mean NFLD score for each color was statistically compared by Steel-Dwass tests. The intra- and inter-rater agreement of the two graders was assessed by weighted kappa analysis.

#### Evaluations of the number of NFLDs

To compare the number of NFLDs detected in the fundus photographs and/or the MC-SLO images, the number of NFLDs was compared in cases where NFLD could be confirmed in the images of both instruments. Two retinal specialists (SS, NK) counted the number of NFLDs and compared the numbers by Steel-Dwass tes.

#### Agreements between NFLDs detected by MC-SLO and thinning of RNFL map determined by OCT

It is known that the RNFL is thinner in the sector corresponding to the NFLD in patients with glaucoma [11]. Thus, we studied whether the sector of the RNFL corresponding to the non-glaucomatous NFLDs detected by MC-SLO was thinner. We compared the RNFL map in the macular area taken by 7 × 7 mm macular maps (DRI OCT) and MC-SLO images of blue wavelength which is the most sensitive color channel for non-glaucomatous NFLDs. When a thinner RNFL corresponding with NFLDs was found in the RNFL thickness map, they were classified as “agreed”. These assessments were made by the two examiners (HT, HS).

## Results

### Demographics of patients

The demographics of the patients are presented in Table 1. Thirty-one eyes of 26 patients, 19 men and 7 women, were studied. There were 14 eyes of 10 patients with diabetic retinopathy (DR), 11 eyes of 10 patients with age-related macular degeneration (AMD), 3 eyes of 3 patients with a branch retinal vein occlusion (BRVO), and 3 eyes of 3 patients with an epiretinal membrane/macular hole (ERM/MH). The mean age of the patients was 63.1 ± 11.2 years with a range of 42 to 86 years. The mean refractive error (spherical equivalent) was − 0.72 ± 1.17 diopters, and the mean axial length was 23.1 ± 1.17 mm. The mean best-corrected visual acuity (BCVA) was 0.25 ± 0.37 logMAR units (snellen visual acuity, 20/35). Sixteen eyes were pseudophakic. All patients with DR and BRVO had been treated by retinal photocoagulation. Five eyes with DR and 3 eyes with MH/ERM had undergone vitrectomy prior to this study. In these cases, 3 eyes with DR and 3 eyes with MH/ERM had the inner limiting membrane (ILM) peeled during the vitrectomy.

**Table 1:** Demographics of patients

	DR	AMD	BRVO	MH/ERM	Total
No. of eyes	14	11	3	3	31
Sex (M/W)	7/3	9/1	2/1	1/2	19/7
Age	63.0 ± 11.8	75.6 ± 7.9	58.3 ± 8.7	67.0 ± 5.6	63.1 ± 11.2
Refractive error	−0.61 ± 0.92	0.67 ± 1.2	−0.32 ± 0.82	−1.43 ± 0.75	− 0.72 ± 1.17
Axial Length	23.4 ± 0.72	23.2 ± 0.67	23.3 ± 0.83	22.0 ± 3.16	23.1 ± 1.17
Visual Acuity (logMAR)	0.26 ± 0.28	0.38 ± 0.55	0.12 ± 0.1	0.15 ± 0.15	0.25 ± 0.37
Lens Status (phakic/pseudophakic)	2/12	9/2	3/0	1/2	15/16
Prior photocoagulation	14/14	0/11	3/3	0/3	17/31
Vitrectomy/no vitrectomy	5/9	0/11	0/3	3/0	8/23

### Intra-and inter-rater agreement of NFLD scores of MultiColor scanning laser ophthalmoscope and color fundus images

The kappa index of intra-and inter-rater agreements for each channel of the MC-SLO and CF images were good at more than 0.6 for both (Table 2).

**Table 2:** Intra- and inter-rater agreement of NFLD score for MC-SLO and color fundus images

	MultiColor-SLO				Color fundus images	
	Blue	Green	Red	Merged	Color	Red-free
Intra-rater	0.707	0.643	0.708	0.631	0.790	0.694
Inter-rater	0.693	0.612	0.733	0.668	0.637	0.694

### Comparisons of NFLD scores for blue, green, red, and merged scanned images of MC-SLO

**Fig. 1:**
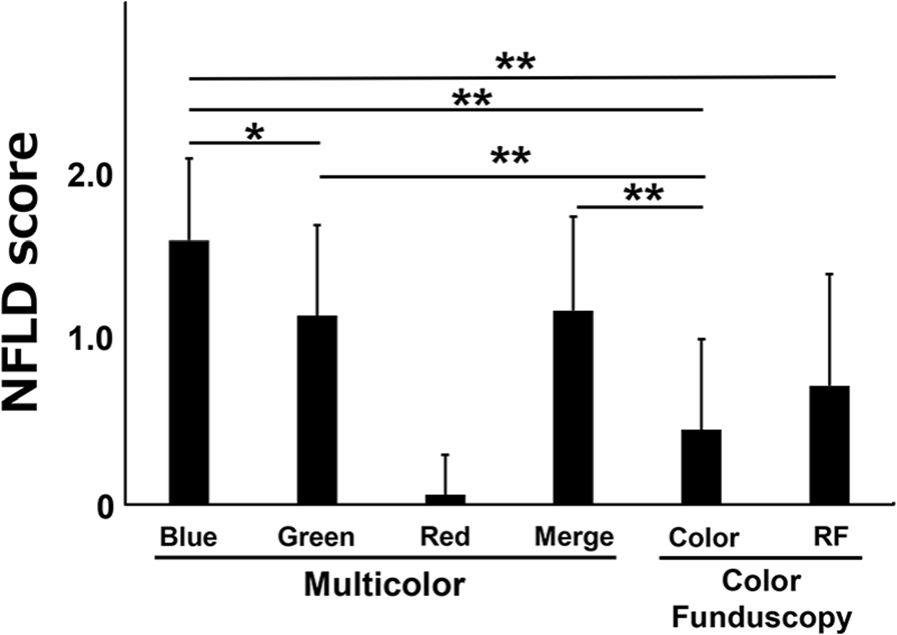
Comparison of NFLD scores by MultiColor scanning laser ophthalmoscope (MC-SLO) and color fundus images. The mean NFLD score was 1.58 ± 0.49 for the red, 1.13 ± 0.54 for the green, 0.06 ± 0.24 for the red, and 1.16 ± 0.56 for the merged MC-SLO images. The NFLD score for the blue wavelength was significantly higher than that for the green and red wavelengths (*P < 0.05 and **P < 0.01, respectively) but not for that of the merged image. The NFLD score for the red MC-SLO was significantly lower than all other images

**Fig. 2:**
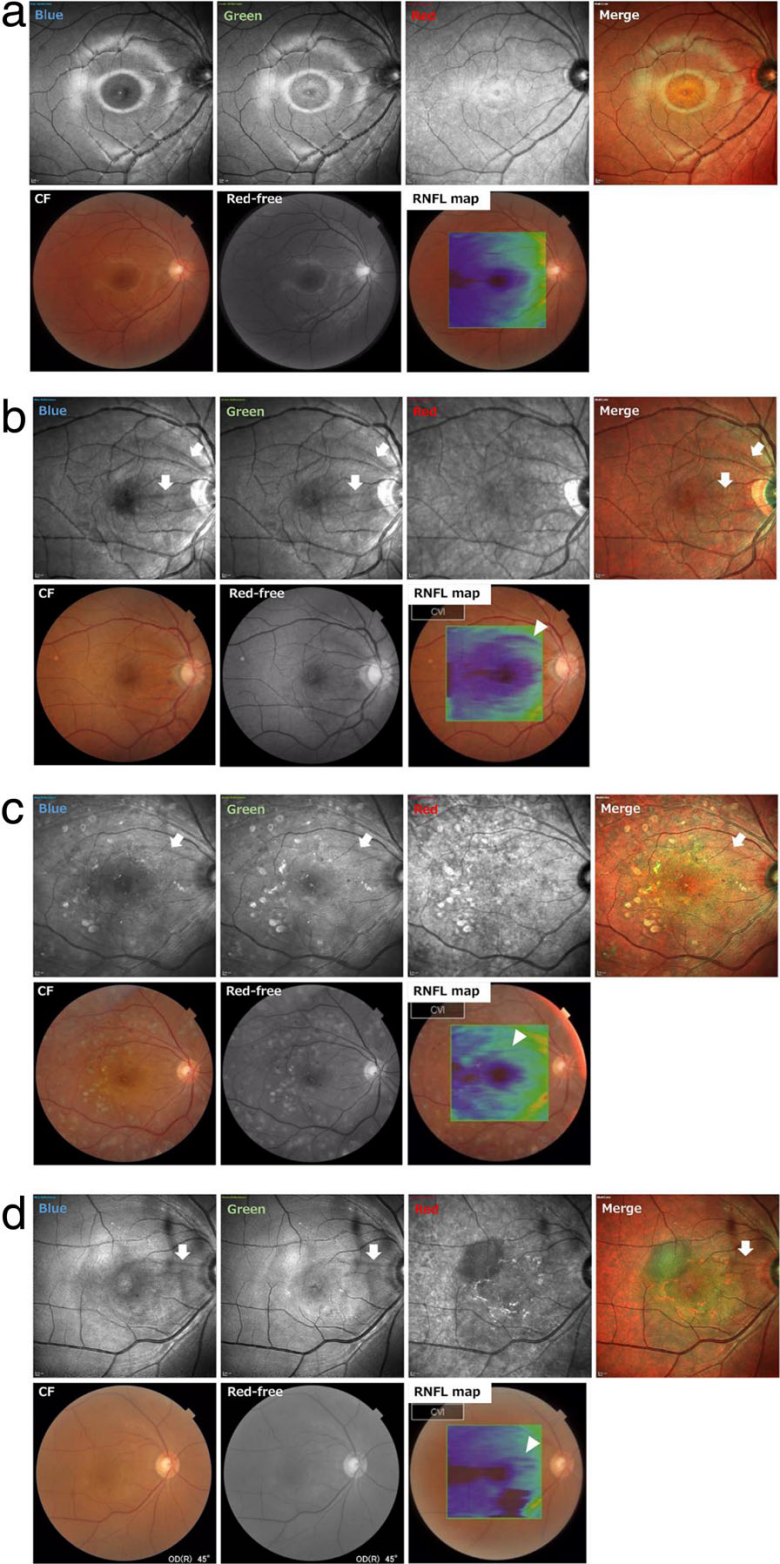
Representative cases. *Case 1*(**a**)*. Healthy right eye of a 28-year-old man. Case 2*(**b**)*.* Right eye of a 73-year-old man with an idiopathic macular hole. Images were taken a month after vitrectomy with ILM peeing. There were two NFLDs in the blue, green and merged images which were not found before the surgery (arrows). Upper NFLD is barely detectable in color and red-free DRI images. *Case 3*(**c**)*.* Right eyes of a 61-year-old woman with proliferative diabetic retinopathy. The eye had had panretinal photocoagulation 7 years earlier. A NFLD was found in blue, green wavelength and merged image (arrows). It was undetected by color and red-free image. *Case 4*(**d**)*.* Right eye of a 69-year-old man with polypoidal choroidal vasculopathy. The eye had been treated with aflibercept injections 6 times and no history of laser photocoagulation. There is a NFLD that was detected in the blue and green (arrows) images but not in the merged image, color and red-free image

Comparisons of the NFLD scores for each image obtained by MC-SLO are shown in Fig. 1. In the healthy subjects, we studied 13 men and 10 women with an average age of 32.2 years (range: 20–45 years old). The average refractive error was − 1.97 ± 1.73 diopters and axial length was 24.2 ± 2.06. In these normal eyes, we did not detect any non-glaucomatous NFLD in all eyes. Representative case of healthy eye was shown in Fig. 2a.

The non-glaucomatous NFLDs were detected by blue wavelength in all cases and 90.3% of NFLDs were detected by green wavelength and merged images and 6.5% by the red wavelength. The mean NFLD score was 1.58 ± 0.49, 1.13 ± 0.54, 0.06 ± 0.24. and 1.16 ± 0.56 for the blue, green, red wavelengths and merged images, respectively. The NFLD score for the blue wavelength was significantly higher than that for the green and red wavelengths (*P* < 0.05 and *P* < 0.01, respectively) but not the merged image. The NFLD score for red was significantly lower than all other images. The mean AL was not significantly correlated with the NFLD score for each wavelength of the MC-SLO images (data not shown).

### NFLD scores of color fundus images

NFLDs were detected in the color images in 12 eyes (38.7%) and in the red-free images in 16 eyes (51.6%). The NFLD score of the color images was 0.45 ± 0.54 and that for the red-free images was 0.71 ± 0.67 (*P* > 0.05). The NFLD scores of the color images were significantly lower than that for the blue, green, and merged MC-SLO images. The NFLD score for the red-free image was significantly lower than that for the blue MC-SLO images. The mean AL was not significantly different among the NFLD scores for the color and red-free images (data not shown).

### Number of NFLDs in MC-SLO and color fundus images

**Fig. 3:**
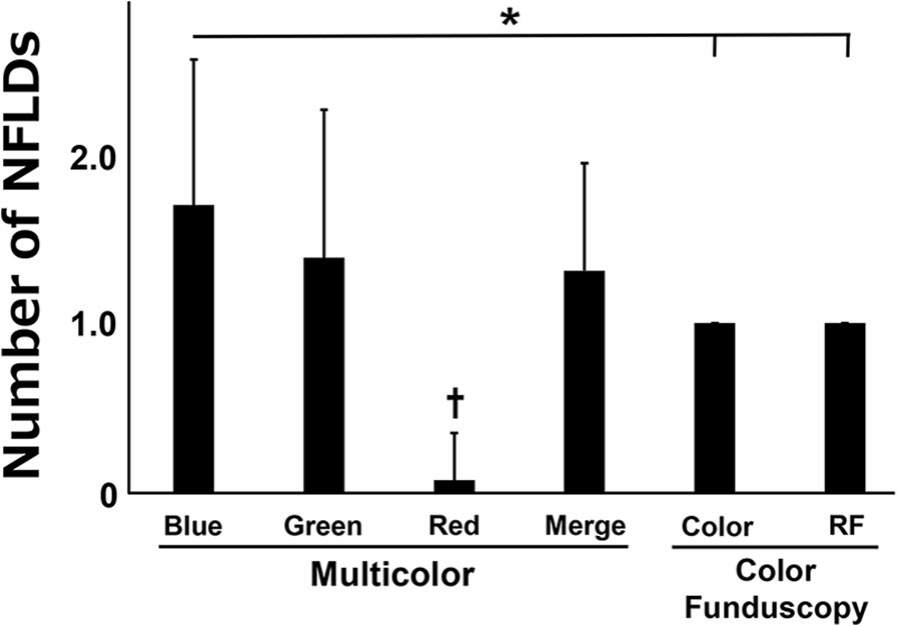
Number of NFLDs in MC-SLO and color fundus images. Number of non-glaucomatous NFLDs in each images were compared in 13 eyes of 13 cases whose NFLD were detected in both the fundus photographs and MC-SLO. There was a statistically significant difference between the number in the blue images of the MC-SLO and the color fundus/RF images (*P < 0.05). †: An average number of NFLDs in red images was significantly lower than that in the other groups (*P* < 0.01)

NFLD were detected in both the fundus photographs and MC-SLO images in 13 eyes of 13 cases. The average number of NFLDs in the MC-SLO images was 1.30 ± 0.63 in the merged images, 1.69 ± 0.85 in the blue SLO images, 1.38 ± 0.63 in the green SLO images, and 0.08 ± 0.27 in the red SLO images. In the color fundus photographs and red-free images, only one NFLD could be confirmed in each case (Fig. 3). There was a statistically significant difference between the number in the blue images of the MC-SLO and the color fundus/RF images. A representative case is shown in Fig. 2b; two NFLDs can be seen in the blue and merged images of MC-SLO, but only one can be barely seen in the fundus photograph/RF.

### Agreements between thinner RNFL and NFLD detected by MC-SLO

The RNFL thickness maps determined by DRI-OCT were available for 26 eyes. In 23 eyes, a thinner RNFL corresponding with the NFLD detected by SLO with blue wavelength was found (88%).

### Representative cases

Three representative cases are shown in Fig. 2a-d.

1. Healthy right eye of a 28-year-old man (Fig. 2a).
2. This is the right eye of a 73-year-old man with an idiopathic macular hole (Fig. 2b). The images were recorded one month after vitrectomy with ILM peeing. There are two NFLDs visible in the blue, green, and merged MC-SLO images which were not detected before the surgery (arrows). The upper NFLD was barely visible in the DRI color and red-free images. Note that the RNFL map showed that the RNFL was thinner in the peripapillary sector corresponding with upper NFLD (arrowhead).
3. The images of the right eye of a 61-year-old woman with proliferative diabetic retinopathy are shown in Fig. 2c. The eye had had panretinal photocoagulation 7 years earlier. A NFLD was detected in the blue, green, and merged images (arrows). It was not detected by the color and red-free DRI images. The sector of the RNFL corresponding with NFLDs appeared to be thinner (arrowhead).
4. The images of the right eye of a 69-year-old man with polypoidal choroidal vasculopathy are shown in Fig. 2d. The eye had been treated with aflibercept injections 6 times but had no history of laser photocoagulation. A NFLD can be seen in the blue and green MC-SLO images (arrows) but not in the red and merged images. The color and red-free DRI images also do not show the NFLD. The retinal nerve fiber layer corresponding to the NFLDs is thinner in the sector corresponding to the NFLD (arrowhead).

## Discussion

Our results showed that non-glaucomatous NFLDs can be detected in the images obtained by MC-SLO. The rate of detection was higher in the blue and green images of the MC-SLO than in the color and red-free images obtained by conventional fundus photographs. These findings suggested that MC-SLO is more useful than the CF in detecting non-glaucomatous NFLDs. In addition, the MC-SLO images were clearer than that of the CF images because of the better resolution of 3.5 μm/pixel which is comparable to that of the B-scan OCT by Spectralis HRA2 (Hidelberg Engineering) [12, 13]. The MC-SLO was also useful because it had three different wavelengths which can be used to obtain SLO images at different depths of the retina. These features result in the superiority of MC-SLO in detecting NFLDs than the CF obtained by conventional fundus photographs.

The NFLDs in glaucomatous eyes are due to a thinning of the RNFL layer [5]. Eighty-eight percent of our cohort had RNFL thinning corresponding to the NFLDs detected in the MC-SLO images. This suggests that the NFLDs are not artifacts but are likely to be pathological changes of the retina.

The pathology of the non-glaucomatous NFLDs have been discussed in eyes with diabetic retinopathy and Bechet’s disease. These studies reported that the non-glaucomatous NFLDs were probably caused by a focal ischemia in the RNFL due to sclerosis associated with the primary disease [6, 7, 14]. Most of the patients in our study had a systemic disorder such as hypertension or diabetes mellitus. In addition, the retinal diseases in our cases, DR, AMD, and BRVO, are associated with disorders of the retinal microcapillaries [15–17]. This is important because these retinal disorders have been shown to have NFLDs [6, 18]. Thus, focal ischemia due to microvasculature damages in the RNFL layer by systemic and retinal conditions may be one of the causes of the non-glaucomatous NFLDs.

Another possible cause is that they are the effects of the treatment of the underlying disorders. Panretinal photocoagulation and ILM peeling during vitrectomy can cause a thinning of the RNFL [19–22]. All of our patients with DR and RVO had a history of photocoagulation, and all the patients with ERM/MH had undergone vitrectomy with ILM peeling. Thus, the non-glaucomatous NFLDs might be due to these treatments.

NFLDs were detected more frequently by blue and green wavelength scanned lights than with the merged or red wavelength lights. This is reasonable because the RNFL is located on the inner part of the retina where the blue light can penetrate. The NFLD score of the merged images was significantly lower than that of the blue images (Figs. 1 and 2c). The merged images contain red wavelength which makes the NFLDs less visible than monochromatic images by blue or green wavelengths. Thus, not only merged images but single blue and green images should be examined when searching for NFLDs with the MC-SLO.

Red-free scanning lights have been used to emphasize the NFLDs in color fundus images. However, little is known about the ability for the red-free lights in detecting NFLDs between color and red-free images. Our results showed that the detection rate of NFLD by red-free image is better than color images but not significantly better than that of color the images (51.6% by red-free v.s. 31.7%). In general, red-free images are generated just by adjustment of the color component of the recorded color images. Thus, the contrast of the NFLDs in red-free images can be emphasized and the contrast is higher than that of the color images but the detection rate of NFLDs by red-free images could not be significantly changed to be better than that of color images.

In general, ophthalmoscopic examinations and color fundus images are the main methods to screen for optic disc disorder. However, they are not effective in detecting non-glaucomatous NFLDs because the optic discs appear normal unlike glaucoma patients. Thus, the higher detection ability of NFLDs by MC-SLO is helpful for retina specialists who tend to pay attention to the retinal condition rather than the glaucomatous findings.

There are limitations in this study. First, this was a retrospective study with a relatively small number of patients. Although the BRVO and ERM groups had fewer cases, we did not include them in the comparisons among the subgroups. Thus, we believe that this limitation did not have a big influence on the results and conclusions. Second, because we studied the patients in a University Hospital, our patients might have more severe disease than patients in primary care facilities. Third, we did not have detailed perimetric and circumpapillary RNFL thickness data by OCT which might have provided us with additional evidence. The relationships between non-glaucomatous NFLDs and these findings should be assessed in future studies. Evaluations of the RNFL thickness by OCT is more accurate in finding glaucomatous changes. However, in the cases without any abnormalities of the optic disc, it appeared that there was no motive to evaluate the RNFL thickness by OCT. Therefore, using MC - SLO for screening of non-glaucomatous NFLD instead of the fundus photographs allowed us to detect the abnormalities earlier.

## Conclusions

Images obtained by MC-SLO are better in detecting NFLDs than those obtained by conventional color fundus photographs in eyes with retinal diseases. The MC-SLO images provides us more detailed information including abnormalities of the RNFL than the color fundus images. MC-SLO should be a more useful method to screen for retinal diseases than CF.

## Abbreviations

AMD: Age-related macular degeneration
BCVA: Best-corrected visual acuity
BRVO: Branch retinal vein occlusion
CF: Color fundus images
DR: Diabetic retinopathy
ERM: Epiretinal membrane
ILM: Inner limiting membrane
MC-SLO: MultiColor scanning laser ophthalmoscope
MH: Macular hole
NFLDs: Nerve fiber layer defects
OCT: Optic coherence tomography
RGCs: Retinal ganglion cells
RNFL: Retinal nerve fiber layer
RPE: Retinal pigment epithelium
